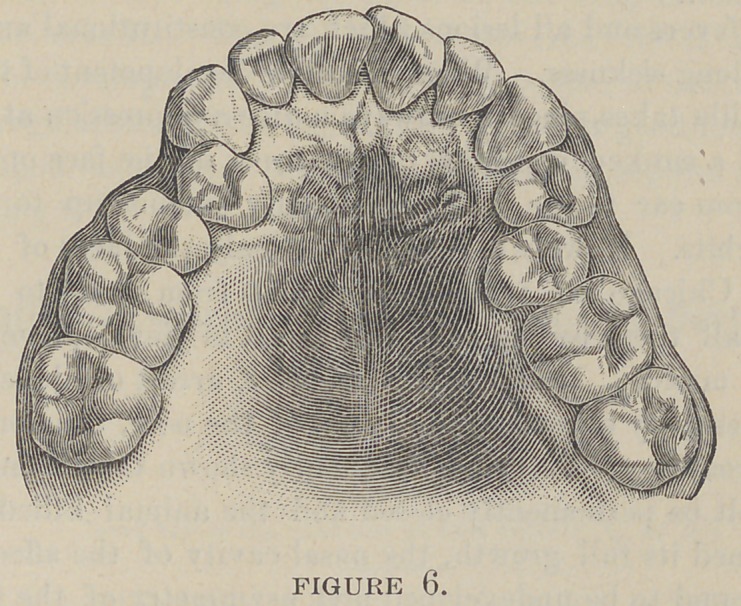# Mouth Breathing Not the Cause of Contracted Jaws and High Vault

**Published:** 1891-12

**Authors:** Eugene S. Talbot

**Affiliations:** Chicago


### Mouth Breathing not the Cause of Contracted Jaws and High Vault.

BY EUGENE S. TALBOT, M.D., D.D.S., OF CHICAGO.

Read before the Section of Laryngology and Otology at the forty-second annual meeting of the American Medical Association at Washington, D. C., May, 1891.

Mouth breathing was not known among the early races, the present pure races or modern uncivilized races, neither are deformities of the jaws and teeth. You will all admit that mouth breathing is becoming a very common occurrence among our own people, and so are also irregularities of the jaws and teeth. It stands to reason then, that the causes which will produce the one must necessarily, in many cases, produce the other.

In an otherwise able article upon the subject of "The Influence of Adenoid Hypertrophy at the Vault of the Pharynx upon the Development of the Haid Palate," read before the New York Odontological Society, November 19, 1890, by Dr. D. Bryson Delavan, the author speaks of mouth breathing as a cause. He says: "The mouth breathing habit compels the constant dropping of the lower jaw, which hanging by the cheek from the superior maxilla, causes constant pressure upon the upper jaw. This produces flattening of the lateral alveolar arches and shortening of them, in consequence of which there is not sufficient space for the eruption of the canines when they are due, and they therefore grow forward."

Other authors mention that sleeping with the mouth open produces tension of the buccinator muscle, this causing the jaws to contract, and they suggest different theories by which this pressure brings about the peculiar form of deformity. There are also very able gentlemen (specialists) teachers in our medical colleges, who are constantly bringing this theory before the students as a cause. This teaching has a tendency to defeat scientific investigation in the direction of ascertaining the real causes of the true condition found in obstruction of the nasal passages by assuming to place the real fact, namely, mouth breathing, as the cause. The students take it for granted that this is the cause and the only cause for this condition.

Let us look at a few facts as they have been presented to me in the constant study of the deformities of the jaws and teeth for the past fourteen years ; and you, gentlemen, shall be the judges whether mouth breathing has any thing to do with contracted arches or not. In the first place let us glance at the parts involved. The superior maxillary bones are united at the median line. The outer surfaces have upon their border an alveolar process. Gray speaks of these two structures as one bone, the superior maxillary bone ; but from the function, structure and position of the alveolar process in its relation to the maxillary bone proper, they should be described as separate and distinct bones. The maxillary bones proper are made up of dense, compact tissue, and are so arranged as to resist force. The outer surface of the bone is fortified and supported by the malar process, which is situated midway between the maxillary process and the canine eminence at the first permanent molar. At the canine eminence we have the strong, thick plate of bone extending from the bridge of the nose to the alæ, the mesial portion forming the outer surface of the nasal cavity. We also observe that the nasal septum is situated at the centre of the nares and is attached to the maxillary bone at and along the place of union of the two halves of the maxillary bone. If a saw was passed through from one canine fossa to the other we should see that it involved the strong pillar of bone which goes to make up the outer surface of the nasal cavity. This strong pillar of bone is situated just at the point of the permanent location of the cuspids ; this, together with the nasal septum, form a strong support to the hard palate. The maxillary bones are for the attachment of muscles and the resistance of force in masticating food. The hard palate does not assume the normal shape until the twelfth year, or after the teeth are all in position. The vault may be high or low, ranging from one inch above the margin of the alveolar process, between the second bicuspid and first permanent molar (which is the highest vault I have seen) down to one-quarter of an inch from the same point, which is the lowest vault I have observed. In either case they are normal, each variety depending upon the shape of the bones of the head for its peculiar form. The alveolar process, on the other hand, is made up of soft, cancellated structure, and is solely for the purpose of protecting the germs of the teeth before they have erupted, and it also supports the teeth after they are in place in the jaw. From the time the teeth make their first appearance until they are lost, the alveolar process has developed and been absorbed three distinct times. The alveolar process then, being solely for the protection and support of the teeth, it stands to reason that the position and shape of the alveolar process depend upon the location of the teeth. The bone proper, therefore, as we shall see later, is not influenced to any great extent by the movement of the teeth. The buccinator muscle is composed of striated muscular fibres and is, therefore, under the control of the will. It is penniform in shape. It has its origin and insertion along the body of the jaws, above the alveolar process on the upper jaw, and below the alveolar process on the lower. It extends from the first bicuspid tooth anteriorly to the wisdom tooth posteriorly. The centre of the muscle would, therefore, be in one direction on a line with the grinding surface of the teeth, and in the other direction at the first permanent molar. Its function is for the purpose of compressing air in the act of blowing, whence its name is derived, and also for the purpose of carrying and holding the food under the teeth during mastication. There are many cases of contracted arches where mouth breathing does not exist. There are also many cases of normal arches where it does exist. As all are aware, mouth breathing frequently commences very early in life; contracted jaws, on the other hand, never commence to form until the seventh or eighth, and in most cases the tenth year, except in cases of monstrosities, or from traumatic causes. When these conditions exist they are wholly unlike the usual contracted arches and can be diagnosticated at once, and therefore should not enter into this discussion. Contracted arches are of two kinds—V (Fig. 1) and saddle (Fig. 2)—all the other varieties being modifications and blendings of these two. It is apparent to every one that the

FIGURE 1.

cause which produces the one does not produce the other. My observation has been that there are two-thirds more V and saddle-shaped arches among the low vaults than among the high vaults, taking $\frac{18.77}{32}$ of an inch as an average, but where one of these deformities exists with a high vault it is always more marked, for the reason that in the high vault the alveolar process is high and thin, and the teeth are more easily carried in one direction or the other, with very little resistance. In the V-shaped arch commencing at the first permanent molar, there is a gradual narrowing of the teeth and alveolar process toward the median line, where the incisors may reach a point or may stand in their normal position to each other. Invariably there is a protrusion of the teeth and alveolar process, and not the jaw. On the other hand, in the saddle-shaped arch the bicuspids are carried inward and the deformity is invariably situated between the first permanent molar and the cuspid. Unlike the V-shaped variety the anterior teeth and alveolar process never protrude in this class of deformities. The contracted hard palate is always associated with the V-shaped variety, and in most cases extends backward to the second bicuspid. It is never seen with the saddle-shaped variety. The high vault is never seen in the first set of teeth, nor does it develop until the second set are all in place, which is at the twelfth year. The vault commences to slope slightly from the neck of the incisor until it reaches a line

FIGURE 2.

drawn across the roof of the mouth from the first right bicuspid to the first left bicuspid, and then it gradually or abruptly slopes upward until a line is reached which is drawn across the jaw from the anterior surface of the opposite permanent molar. From this point posteriorly to the soft palate the dome is usually on a level; occasionally we see a slight depression and occasionally a slight elevation, but these are so slight as to escape notice unless one were looking for the peculiarity. In mouth breathing the lower jaw usually drops only sufficient for the passage of the same volume of air as would pass through the nasal cavities, which is only about one-half inch. Old people often sleep with the mouth open and frequently to the fullest extent, but these deformities of the jaws and teeth never occur after the eruption of the teeth, say at the twelfth or fifteenth year. When one opens his mouth he is conscious of a tension of the orbicularis oris, but not of a pressure of the buccinator, no matter how wide it may be opened. This muscle being under the control of the will is always passive except in the act of blowing or eating, therefore contraction during sleep is wholly out of the question. As the buccinator muscle extends anteriorly to the first bicuspid only, it can produce no effect upon the V-shaped variety of deformity, in which is also found the contracted vault. Therefore, the only deformity that is likely to be produced is the

FIGURE 3.

saddle-shaped variety, which is out of the question for reasons which I shall explain later. The orbicularis oris muscle can not produce the contraction because when the mouth is open the pressure exerted on the six anterior teeth is backward. Thus the teeth are carried in the opposite direction from that which must be taken to produce this deformity. Again, the pressure is just as great upon the incisors as upon the cuspids, thus holding them in place. More force is exerted by the orbicularis oris upon the six anterior teeth when the mouth is open than can be exerted (if it were possible) by the buccinator muscle, which would tend to hold the anterior teeth in place. For years it has been demonstrated by dentists in regulating teeth that it is very rare for the apices of the roots of teeth to move when pressure is brought to bear upon the crowns of teeth for the purpose of regulating them. This being the case, teeth having long roots like the cuspids are less liable to move than teeth with short roots like the lateral incisors and bicuspids. Since in the moving of a tooth the greatest change which takes place is at the neck, it stands to reason that the greatest absorption and deposition of bone takes place at that point. The roots of the cuspid teeth are larger and longer than any other teeth in the jaw: unlike other teeth the germs are situated considerably highter and farther

FIGURE 4.

toward the outside of the alveolar process, so that when they come close into position they diverge from the apices to the crowns, while all the other teeth stand nearly, or quite, perpendicular, thus showing that the roots of these teeth do not influence the hard palate. I have shown that the first permanent molar and the teeth posterior to them are never involved. I have also shown that the centre of the muscle in both directions is located at this tooth. How is it possible, since all the teeth are covered by the muscle upon one side, that half are carried inward and the other half remain normal? Again, if mouth breathing is the cause of the contraction, both sides must contract alike, and the deformity must be uniform upon both sides, which is never the case. Muscles do not contract to a degree sufficient to produce the pressure necessary to produce a deformity. It is inconsistent with our knowledge of the influence exerted by muscular structure in other parts of the body. Some of the muscles of the chest exert much more pressure in respiration than it is possible for the buccinator to do during sleep, yet no one would expect to find the ribs modified by this process. The pressure of the tissue upon the crown of the teeth is not sufficient to affect the alveolar process through the roots of the teeth, but even if it could modify those spongy structures its force would stop there, and would not extend to the osseous vault, bending it out of shape. In most of these cases the superior maxilla and the diameter of the alveolar process and teeth is very much smaller than the inferior maxilla, alveolar process and teeth; in such cases the muscles and cheek could not reach the teeth and alveolar process upon the upper jaw. This is always the case in the worst forms of irregularities. The changes which take place in bone are not a bending in at one place and forcing out at a weaker point to compensate for the space lost, but are an absorption and deposition of bone at the point of pressure. And even if such were the case, the strong pillar of bone situated at the very point of contraction of the alveolar process, together with the nasal septum, both form a strong bulwark for the resistance to the pressure which is situated quite a distance from the top of the vault. Again, it would be as impossible to produce pressure sufficient to break the dental arch as it would be for the weight of a building to break the arch of a door or window. The tongue exerts a much greater force in the act of swallowing and would prevent the inward movement of the teeth if so slight a pressure as the muscles of the cheeks were the cause of the deformity. For the sake of argument let us suppose it were possible for the buccinator muscle to produce this contraction ; we should then expect to find the modification of the osseous structures uniform. This would shut out semi-V (Fig. 3) and semi-saddle-shape arches (Fig. 4) entirely, and a majority of other irregularities of the teeth in which there is bilateral asymmetry, for however much one would incline to the prevalent theory, no one would dare to assert that the muscle will act on one side of the mouth, while that on the opposite side remains passive. Partial V- (Fig. 5) and partial saddle-shaped arches (Fig. 6) make it still less plausible. In these we meet with sudden bends inward where only one or two teeth may be involved, which could only be produced by a centralization of force on one given point or fibre of muscle, a peculiarity of function that has never

FIGURE 5.

yet been ascribed to muscles. The muscle being penniform in shape it would be impossible for one or two fibres of the muscle to exert its influence upon a bicuspid. It would naturally lap over two or more teeth. Lastly, if the buccinator acts as all muscles uniformly throughout its extent of contraction, below its median line it is just as efficient in producing a narrow contracted arch as in its upper portion, and we should expect to find the lower maxilla contracted whenever the upper one is, which is contrary to facts. A V-shaped arch can never occur upon the lower jaw if the teeth articulate normally because these teeth strike inside of the upper and are usually prevented from moving forward. A saddle, partial saddle and semi-saddle arch may occur on the lower jaw but these deformities are not often seen. When they do occur they are the result of improper occlusion with the teeth of the upper jaw. We always observe in semi-V and partial V-shaped arches that the alveolar process is contracted upon the side of the deformity. If one side is contracted more than the other we shall observe that the alveolar process is contracted in proportion to the amount of the defor-

FIGURE 6.

mity; the vault on that side is not carried up beyond the other side, which is normal. In the saddle, semi-saddle and partial saddle-shaped arches we find the alveolar process built up about the teeth in the precise uniformity to the nature of the shape of the arch. If we take 3,000 models of the upper jaw and arrange them in groups according to the forms here represented and then examine very closely the arrangement of the teeth in each group, we will be unable to find any two alike in either group; thus showing that an external force acting upon the jaws from the outside could not possibly be the cause. If it were possible all the models of one variety would resemble some exact form. Dr. Delavan says that "The prominence of the anterior region of the alveolar arch is still further increased by the projection forward of the superior maxilla at this point, and of the upper teeth." The doctor is quite mistaken as regards the "projection forward of the superior maxilla." The maxillary bone never protrudes in front in this class of cases, it is only the alveolar process which is carried forward by the projecting teeth. The only issues involved in these deformities are the teeth on the one hand, and the alveolar process on the other.

In most cases the cause of these deformities is arrest of development of the maxillary bone. This condition is due not only to hereditary influence, but also to direct causes such as the eruptive fevers and all lesions which are constitutional and which produce long sickness. When arrest of development of the superior maxilla takes place we always notice a depression at the alæ nasi, and a sunken condition of the bones of the face on the line drawn from ear to ear, and occasionally extends up to the floor of the orbits. If we will examine closely the faces of an audience in Chicago we will observe that from forty to fifty per cent. of all these people have this arrest of development of the superior maxilla. Such being the case, arrest of development must necessarily extend to the bones of the nose, thus producing mouth breathing. Ziem has frequently shown that if one nostril of a rabbit be permanently closed and the animal killed after it has attained its full growth, the nasal cavity of the affected side will be found to be undeveloped and asymmetry of the face will take place. Arrest of development of the bones of the nose and hypertrophy of the bones and mucous membrane will ensue as a result. A good illustration of hypertrophy of mucous membrane from want of use is observed by dentists when the gums puff up, thicken and extend one-half to three-fourths of the length of the teeth from want of brushing. It would be useless for any one to say that mouth breathing is the cause of one case of V-shaped arch in every twenty and that some other cause produced the rest of the deformities. We must have a law which will work in all varieties of contracted arches as well as the V-

shaped, which variety constitutes a very small percentage of the whole. I have watched the development of these different varieties for the past fourteen years, have taken impressions of the mouths of some of the most marked cases every three months and compared them. I have also produced most of these forms in the movement of the teeth for the purpose of correcting deformities.

I regret that it will be impossible at this time to show how these different forms of irregularities of the teeth are produced, but they are nicely described and illustrated in my work upon "Irregularities of the Jaws and Teeth, and Their Treatment." I will, however, say that they are caused by the long diameter of the dental arch being too great for the long diameter of the superior maxilla. Having then discovered the cause (that of arrest of development of the maxillary bones) of contracted jaws and irregularities of the teeth, have we not a good foundation to work upon to discover the cause of deflected septum and mouth breathing?

## Figures and Tables

**Figure 1. f1:**
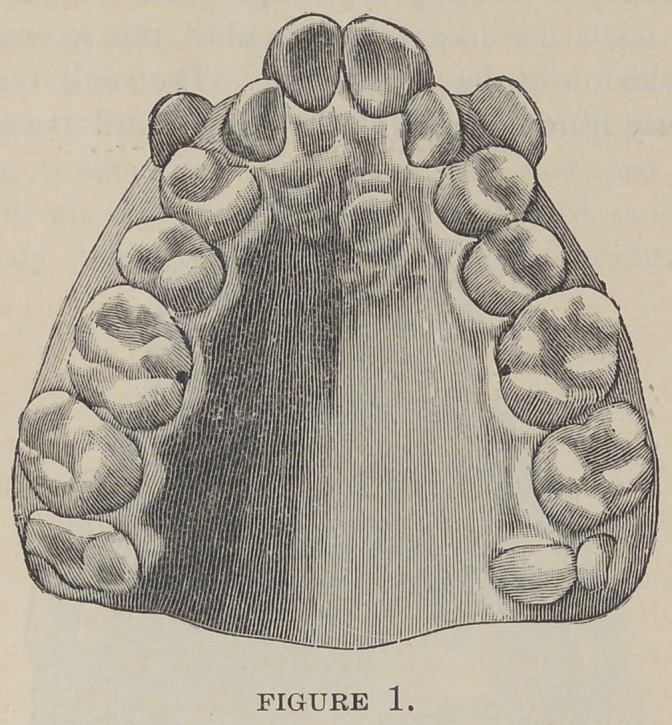


**Figure 2. f2:**
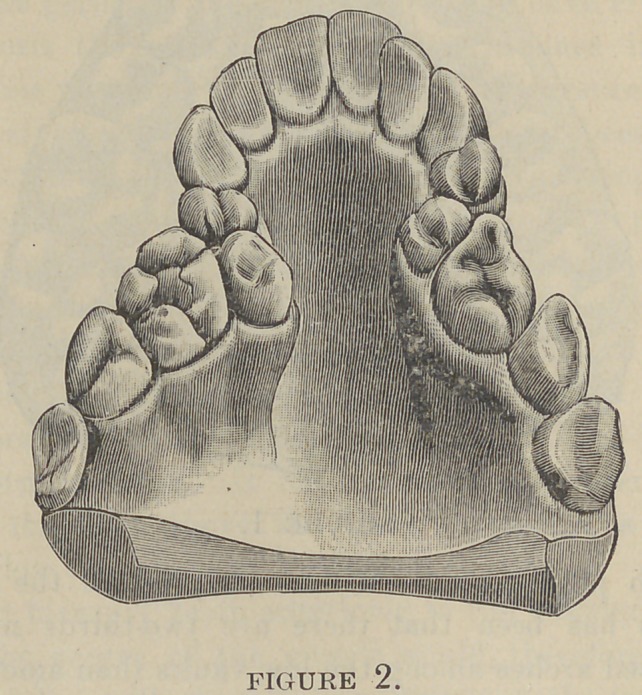


**Figure 3. f3:**
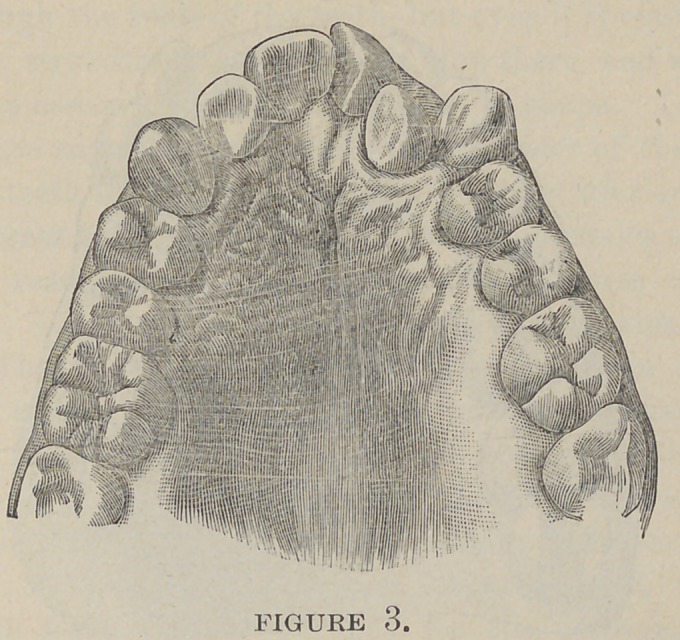


**Figure 4. f4:**
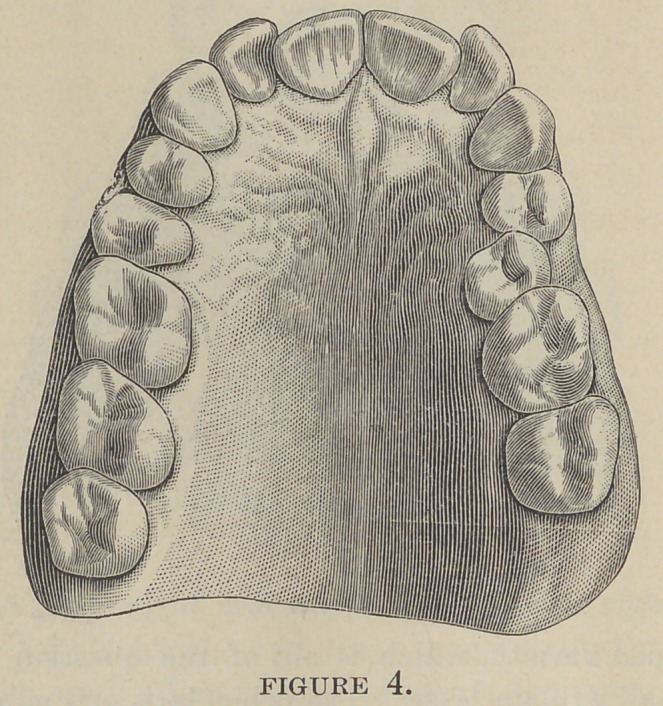


**Figure 5. f5:**
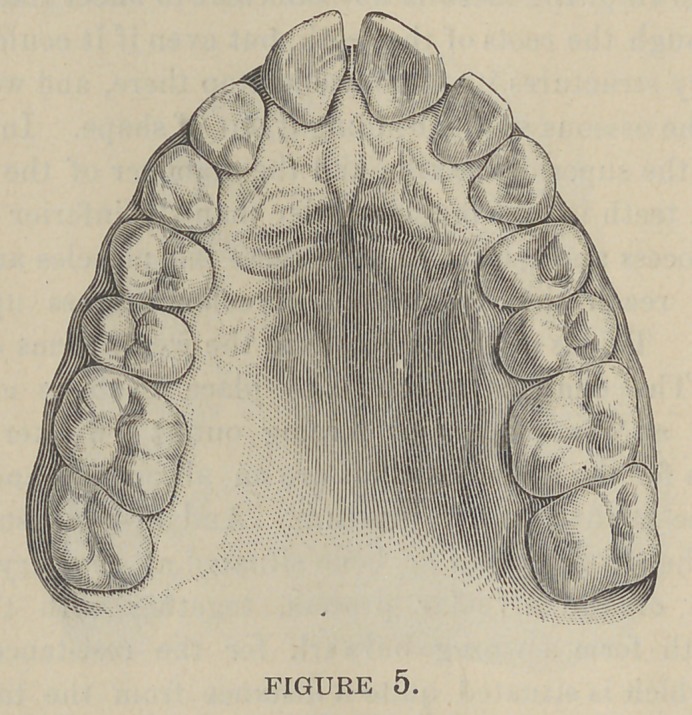


**Figure 6. f6:**